# Testing the Implementation of the PAIN-CPG-EIT: Protocol for a Randomized Clinical Trial

**DOI:** 10.2196/72985

**Published:** 2026-09-30

**Authors:** Barbara Resnick, Elizabeth Galik, Rachel McPherson, Nayeon Kim, Shijun Zhu

**Affiliations:** 1 University of Maryland, Baltimore, 655 West Lombard Street, Baltimore, MD, 21201, United States, 1 4438122735, 1 4435724338

**Keywords:** pain, nursing home, dementia, pharmaceutical, nonpharmaceutical

## Abstract

**Background:**

Pain is a common symptom among older adults in nursing home settings, affecting 30% to 80% of residents living with dementia. Pain is not assessed; the underlying cause is not identified, and treatment is not initiated for a large percentage of residents. Untreated or overtreated pain can lower quality of life, negatively impact function, impair sleep, and increase behavioral and psychological symptoms associated with dementia.

**Objective:**

The purpose of this National Institute of Aging–funded study is to use our theoretically based approach that includes the social ecological model, social cognitive theory, and the evidence integration triangle to translate the use of the Pain Management Clinical Practice Guideline (Pain Management CPG) into nursing home settings and improve the assessment, diagnosis, and management of pain among residents living with dementia. Our theoretically based approach, combined with the Pain Management CPG, is referred to as PAIN-CPG-EIT (Pain-Clinical Practice Guideline-Using the Evidence Integration Triangle).

**Methods:**

The study is a cluster-randomized clinical trial, with communities assigned to receive treatment with the PAIN-CPG-EIT vs pain education only (EO). The nursing homes have been randomized equally into intervention and control groups. The goal is to recruit a total of 300 residents from 12 nursing homes (25 residents from each community). Implementation of the study is being done via 3 cohorts, with 4 communities included in each cohort. The PAIN-CPG-EIT is implemented by a research nurse facilitator and includes 4 components, namely, component I: establishing and meeting with a stakeholder team; component II: education of the staff; component III: mentoring and motivating the staff to address pain; and component IV: ongoing monitoring of pain management in the community. The EO intervention is also implemented by a research nurse facilitator and consists of component II only, which is education of the staff.

**Results:**

Study activities began in September 2023, and recruitment of residents and intervention activities started with cohort 1 in January 2024. Cohort 2 was initiated in February 2025, and cohort 3 was initiated in June 2026 and is currently ongoing. A total of 208 residents have been recruited from the first 10 study sites. All intervention activities have been implemented as intended. The study is expected to be completed by December 2027.

**Conclusions:**

Outcomes are evaluated at baseline, 4 months, and 12 months after implementation of the intervention, and include improvement in the assessment, diagnosis, and management of pain; a decrease in pain; and evidence of more appropriate use of opioids. The findings from this study will provide evidence of the effectiveness of our implementation approach and use of the Pain Management CPG. The theoretically based approach may be useful for the implementation of other CPGs in nursing homes.

## Introduction

### Background

Pain is a commonly reported symptom affecting 22% to 85% of individuals living with dementia in nursing homes [1-3]. Over 50% of these individuals experience chronic pain [1,2]. Pain, however, is not always documented in the assessment of these individuals due to cognitive status and their inability to report pain directly [3-5]. Furthermore, for those with moderate to severe dementia, verbal reporting of pain may not be reliable [6]. Multiple systematic reviews [7,8] concluded that self-report tools should be used to evaluate pain among these individuals, when possible, but observational measures are needed to supplement self-report. Specifically, it is recommended that observational measures should be added when the individual has evidence of moderate dementia based on the Brief Interview for Mental Status (BIMS) scores of 8 to 12 or the equivalent Mini-Mental Status Examination score of 18 [9,10].

Pain has a significant impact on older adults living with dementia. It has been repeatedly noted that pain causes a decline in function [11-14] and quality of life [12,14-22], exacerbates behavioral symptoms associated with dementia, such as aggression, agitation, repetitive vocalizations, withdrawal, depression, and apathy [14,23], contributes to delirium [22,24,25], decreases engagement in daily activities [26], and increases the risk of emergency room visits [27].

Although it is well recognized that there are challenges to the assessment, diagnosis, and management of pain among older adults in nursing homes, particularly for those living with dementia [28], there has been no clear guidance for how to best improve pain management [28]. Recommendations have included the use of clinical guidelines, mandated education, and multifaceted assessment tools. In the United States, the state operations manual for long-term care [29], specifically F-Tag F697, requires that nursing homes ensure that pain management is provided to residents who require such services and that the management is consistent with professional standards of practice, the comprehensive person-centered care plan, and the residents’ goals and preferences. Although these regulations are provided to communities, it is not clear how the communities can best achieve the required outcomes. To help fill this guidance gap, the Post Acute and Long-Term Care Medical Association (PALTMed, previously known as the American Medical Directors Association) developed the Pain Management Clinical Practice Guideline (Pain Management CPG) [30]. The Pain Management CPG was developed by an interdisciplinary team that reviews important issues and questions related to pain, addresses 25 key questions (eg, when are opioids likely to be helpful and what treatments may be effective in various types of pain?), includes 37 clinically relevant and practical tables, and a comprehensive list of 150 references. The purpose of this protocol is to test an approach to help communities implement the Pain Management CPG using a well-established, theoretically based approach. The implementation intervention approach is referred to as the PAIN-CPG-EIT (Pain-Clinical Practice Guideline-Using the Evidence Integration Triangle).

### Theoretical Framework Guiding the PAIN-CPG-EIT

The social ecological model [31] was used as the overall model for factors that can influence the implementation of the Pain Management CPG. This model addresses intrapersonal factors (eg, age, gender, race, and beliefs), interpersonal interactions between staff and residents (eg, listening and demonstrating empathy), components of the environment (uncluttered areas to ambulate, comfortable chairs to sit on, and distractions), and policies (regulations around the use of heat, cold, and music for pain interventions; access to pharmaceutical options) that influence pain among older residents with dementia.

Social cognitive theory was used to provide guidance for how to motivate staff and residents to engage in pain assessments, diagnoses, interventions, documentation of pain-related care, and monitoring of outcomes. It includes self-efficacy expectations, which are an individual’s belief in their ability to complete a pain-related intervention (eg, assess pain in a noncommunicative resident). Outcome expectations focus on the impact of the behavior performed (eg, if I provide nonpharmacologic interventions such as heat, this will help neck pain in this resident). The higher the self-efficacy or the outcome expectation, the more likely the individual will be motivated to engage in the activity. Self-efficacy and outcome expectations can be strengthened by 4 approaches, as shown in Textbox 1 [32-34]. These 2 theories guided the development of the 4 components of the intervention, namely, component I: establishing the stakeholder team and monthly meeting schedule, reviewing the implementation plan, and establishing community goals focused on pain; component II: education of the staff using our established PowerPoint, which includes a review of the Pain Management CPG with a focus on assessment, diagnosis, management, and ongoing evaluation of pain; component III: mentoring and motivating the staff to address pain using the Pain Management CPG and using the weekly email “Tidbits.” The Tidbits, which are emailed to the stakeholder team members, provide information about pain assessment, diagnosis, and management, and the use of motivational techniques to engage staff in addressing pain among the residents. The stakeholder team members are encouraged to share the Tidbits with all staff; and component IV: providing ongoing monitoring of pain management in the community (eg, improved documentation of pain assessment, diagnosis, and management, and better pain relief among residents).

Textbox 1. **Social cognitive theory: sources of information and examples.**

**Actual performance of the activity**

- Staff assessing pain accurately and documenting; providing ice for knee pain
- Resident going for a walk or sitting up to relieve pain from lying in bed

**Verbal encouragement**

- Nurse interventionist acknowledging staff for accurate assessments of pain or implementation of innovative approaches to manage the pain
- Encouraging a resident to listen to some music to distract him or herself from focusing on the pain

**Exposure to role models or self-modeling**

- Highlighting staff who initiate innovative approaches to pain management
- Reviewing with a resident how they managed the pain the previous day and encouraging them to try that approach again

**Eliminating unpleasant sensations associated with an activity and focusing on positive sensations or outcomes**

- Showing the staff it does not take longer to apply ice or heat than to medicate a patient for pain
- Showing a resident that ice or local ointments, such as icy/hot or others, can help to relieve the pain in a joint

Evidence integration triangle (EIT) serves as a guide to the implementation of the 4 components and use of the Pain Management CPG. EIT includes a participatory process, practical evidence-based interventions, and pragmatic measures of progress toward goals, as delineated in Figure 1.

**Figure 1.**
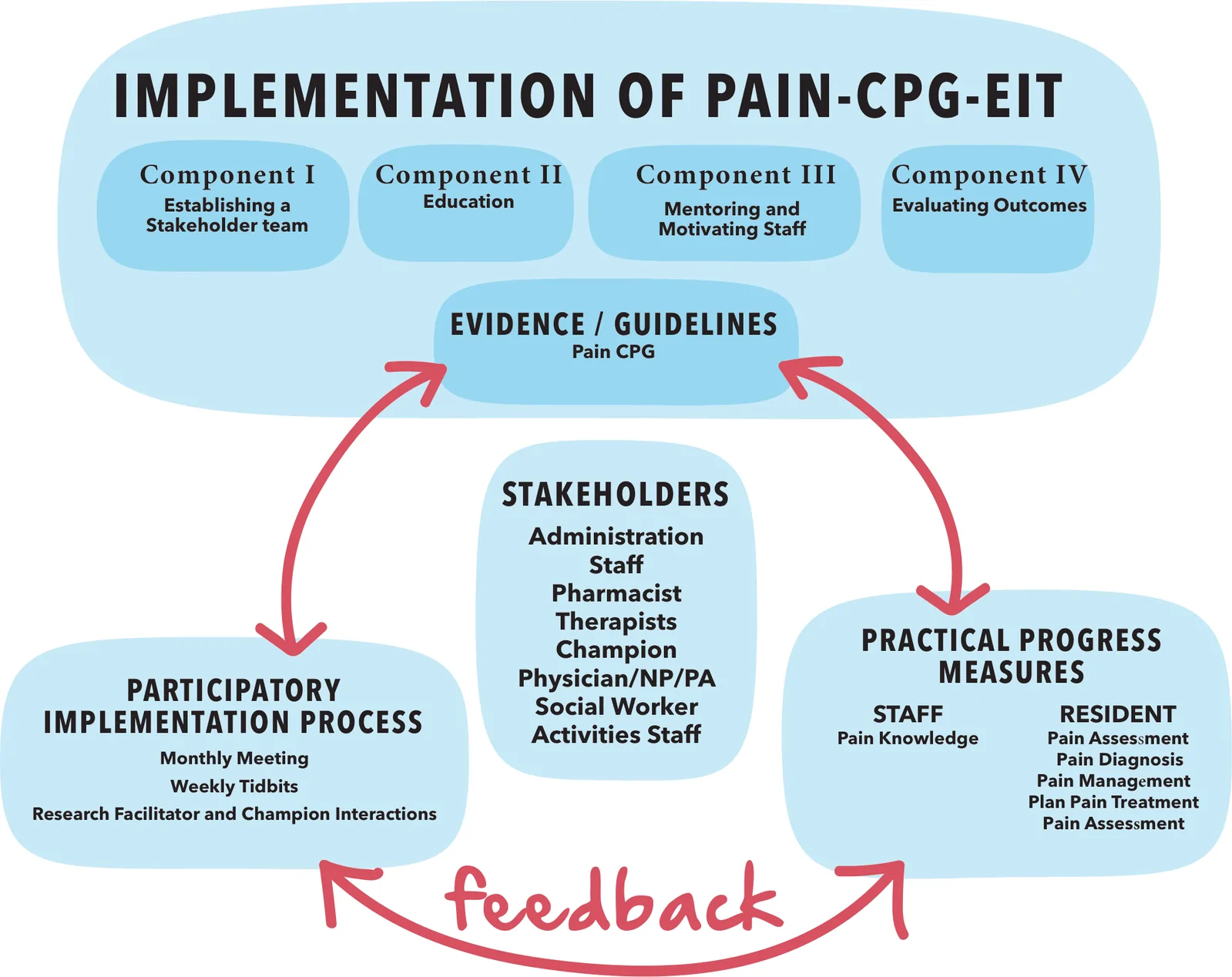
Theoretical approach to PAIN-CPG-EIT. CPG: clinical practice guideline.

### Specific Aims

The specific aims of this study are to (1) test the effectiveness of using the PAIN-CPG-EIT to improve the assessment, diagnosis, and management of pain; decrease pain intensity; and demonstrate appropriate use of opioids among nursing home residents living with dementia; (2) evaluate treatment fidelity related to the use of the PAIN-CPG-EIT based on delivery (all components of the intervention delivered), receipt (all staff demonstrating knowledge of pain assessment, diagnosis, and management as delineated in the Pain Management CPG), and enactment (community goal around pain management achieved); and (3a) test for gender/sex and race invariance in pain measurement based on the PAINAD (Pain Assessment in Advanced Dementia) Scale; and (3b) test for differences in treatment of pain and response to treatment between male and female and Black vs White residents living with dementia. Findings from this study will help to improve the assessment, diagnosis, and management of pain among older adults living with dementia who are experiencing pain. Demonstrating the effectiveness of this implementation approach to using the Pain Management CPG will facilitate future work to disseminate and implement this CPG.

## Methods

### Study Design

The study is using a cluster-randomized design, as testing of the PAIN-CPG-EIT requires a community-wide approach to prevent treatment contamination that might occur if we were to randomize residents within communities. A safety monitor was identified to provide oversight of the study.

### Ethical Considerations

The study was approved by the University of Maryland Institutional Review Board (Protocol HP-00105286). The study was registered at ClinicalTrials.gov (identifier number: NCT05858996; registered on May 16, 2023). All participants and legally authorized representatives consented to participate as per the approved consent form.

### Settings and Residents

A total of 12 communities were randomized to treatment (PAIN-CPG-EIT) or pain education only (EO) using a simple randomization technique [35]. Nursing homes were invited and eligible to participate if they (1) had at least 100 beds; (2) identified a nurse (registered or licensed practical nurse) to be the champion; and (3) were Medicare-certified. Residents are eligible to participate if they (1) live in a participating community; (2) were 65 years of age or older, which was then revised to 60 years of age or older to help with recruitment as there were several individuals 60 years of age or older otherwise eligible to participate; (3) were screen positive for dementia based on a recommended process [36] that includes a score of 0 to 12 on the BIMS [37]; a score of >2 on the AD8 Dementia Screening Interview [38,39]; a score of 0.5 to 2.0 on the Clinical Dementia Rating (CDR) Scale [40]; and lastly a score of ≥9 on the Functional Activities Questionnaire (FAQ) [41,42]. The AD8, CDR, and FAQ are completed by an informant, and all screening measures are reliable, valid, accurate, and pragmatically differentiate those with and without dementia [43]; (4) have evidence of consistent pain at the time of recruitment based on the Minimum Data Set assessment item: How much of the time in the last 5 days have you experienced pain or hurting with eligibility based on the following responses or evidence: occasionally, frequently, or almost constantly, or staff report of pain at the same frequency; or if the resident is receiving nonpharmacological or pharmacological treatment for pain. Residents are excluded if they are (1) admitted to the community for short-stay rehabilitation or other subacute needs (eg, intravenous antibiotics). While initially residents were excluded if they were on Hospice, this was revised in the protocol as we noted that there were individuals who were on Hospice and remaining so for greater than a 12-month period. These individuals would, therefore, be available for 12-month follow-up, and thus it was appropriate to include them in the study. We also increased our eligibility to include those with a BIMS score of 13 to increase the number of potentially eligible participants. Both modifications occurred during recruitment in cohort 1 in our first community. For recruitment, we obtained a list of all potentially eligible residents from a designated staff member and randomly approached each resident until 25 residents per nursing home were recruited by research assistants.

Sample size calculation was based on our primary aim of testing the effectiveness of the PAIN-CPG-EIT to improve the assessment, diagnosis, and management of pain and to decrease pain among nursing home residents living with dementia. For resident-level outcomes, we conservatively proposed the minimum detectable effect size as small-to-medium (Cohen *d*=0.3). Assuming the correlation between repeated measures as *r*=0.7 and the clustering within each community as *P*=.03, a sample of 240 residents (20 residents for 12 nursing homes) would provide sufficient power (*P*>.80) to detect this difference. Based on prior research, we anticipated a 20% loss to follow-up over 12 months [44,45] and no dropout at the community level. Therefore, we planned to recruit a total of 300 residents from 12 nursing homes (25 residents from each community).

### The PAIN-CPG-EIT Intervention

Following randomization, we work with the treatment communities to identify champions and stakeholder team members. We recommend the following stakeholders: a nurse in a leadership position (eg, director of nursing); the designated champions; the nursing home administrator; a physician or medical director, a nurse practitioner, and/or physician associate; a unit nurse; a nursing assistant; activity staff; a social worker; a pharmacist; and rehabilitation therapists. As per community preferences, other team members may also be included. The 4 components of the PAIN-CPG-EIT are provided by a research nurse facilitator working with the champions and stakeholder team.

The research nurse facilitator arranges the first face-to-face and/or Zoom meeting (per the preference of the group) with the stakeholders in each community and implements the 4 components of PAIN-CPG-EIT (Table 1). The research nurse facilitator works 8 hours weekly during months 1 and 2 and then 4 hours weekly starting in month 3, for a total of 12 months, to provide the 4 components of the PAIN-CPG-EIT. Details of the components are provided in Table 1. Briefly, during component I, the first stakeholder team is provided with information about the implementation process, a review of the Pain Management CPG, and participates in a brainstorming session to identify a community goal related to pain. Scheduling of monthly meetings is also done at this time. Component II involves organizing and completing education of the staff using our established PowerPoint. The education is done based on the preferences of the community and is provided in person or via Zoom and recorded for those unable to attend in person. The education provides an overview of the information in the Pain Management CPG with a focus on assessment, diagnosis, management, and ongoing evaluation of pain. Component III involves mentoring and motivating the staff to work with residents to assess pain appropriately by matching the cognition of the resident and his or her ability to complete a verbal or observational pain scale, to evaluate residents for the underlying cause of the pain, to implement interventions to resolve or treat the pain, and to document what was done. We provide weekly email Tidbits (Textbox 2) that include information about pain assessment, diagnosis, and management, and we use motivational techniques to engage staff in pain-related activities with residents (eg, contests to see who has the most innovative approaches to pain management). Component IV focuses on ongoing monitoring of pain management in the community based on the Pain Management CPG. Specifically, this includes evaluating whether residents are being appropriately assessed, whether there is evidence that a diagnosis of the cause of the pain has been established, whether there is a treatment plan in place and implemented, and whether opioids are used appropriately with evidence of the required documentation (see *Measures* section).

**Table 1.** Description of the 4 components of PAIN-CPG-EIT^a^.

Component	Description of activities
Component I: establish and meet monthly with a stakeholder team	Welcome and overview of the project Roles and responsibilities of the research and stakeholder teams Review of the Pain Management CPG^b^ and PAIN-CPG-EIT components Identification of day and evening champions for pain Brainstorming activity and identification of barriers or solutions to the use of the PAIN-CPG-EIT: Brainstorming is a group method for generating ideas or finding solutions to problems. The basic procedure for brainstorming involves identification and delineation of the current approach to assessment and management of pain among residents and asking the group to identify challenges associated with it. The group is then asked to generate solutions to the problems identified. These ideas are written on sticky notes and grouped into themes (referred to as an affinity diagram). The themes may include such things as insufficient knowledge about pain management among staff, or resident or family resistance to treatment. An interrelationship diagram is then developed to determine whether each theme is associated with any of the other themes. The best driver, or root cause, of inadequate pain management is the theme that the community will use to develop its own specific goals for pain assessment, diagnosis, and management. Coordination of monthly meetings (eg, as part of the quality assurance meetings or identifying a time for a separate meeting): The monthly meetings will provide updates to the stakeholder team about challenges or successes related to the use of the Pain Management CPG.
Component II: education of staff	Education will be provided in the preferred format for the community (eg, face-to-face, Zoom, or online and asynchronous). PowerPoint slides and a recording of the presentation have been developed and will be used to provide this education for both current staff and the onboarding of new hires. The education sessions will be kept to 30 minutes and will highlight the following aspects of the Pain Management CPG: (1) different types of pain; (2) appropriate assessment tools to use for each resident (based on cognition or language ability); (3) pain assessment; (4) information to guide pain diagnosis; (5) different treatment options; (6) development of person-centered care plans; (7) documentation and follow-up; (8) raising awareness of differences that may be present in pain assessment, diagnosis, and management between males and females and Black and White residents.
Component III: mentoring and motivating	The research nurse facilitator will work with the champion to establish who will perform what aspects of pain assessment, diagnosis, and management: (1) nursing staff will complete the initial pain assessment (Toolkit Form I or equivalent electronic health record [EHR] data); (2) nursing staff will initiate the evaluation of pain (Toolkit Form II or equivalent EHR data) to help determine the underlying cause of pain; (3) a physician, nurse practitioner, physician assistant, charge nurse, or director of nursing will follow up as needed to complete the assessment initiated by nursing (complete Form II or equivalent EHR data); (4) the interdisciplinary team will establish a plan of care for pain management, which will be integrated into the care plan by nursing (Forms III and IV or equivalent EHR data); (5) nursing staff will evaluate the effectiveness of the plan of care based on the clinical needs of the resident (Toolkit Form V or equivalent EHR data). The research nurse facilitator will work with the champion to help nursing staff ensure that assessments are documented based on the use of Forms I to V or equivalent EHR data and help staff overcome barriers and celebrate successes. Weekly Tidbits will be sent via email to the stakeholder team members. The Tidbits will include updates on pain assessment and management, motivational techniques to engage staff in pain assessment, diagnosis, and management (eg, contests to see who provides the most innovative approach to pain management), and ways to motivate residents to engage in non-pharmacological approaches to pain management, such as physical activity. Pain Management CPG toolkit forms can be uploaded or included in the EHR.
Component IV: ongoing evaluation of resident pain outcomes	The research nurse facilitator will work with the champions to use Table 34 in the Pain Management CPG [30] to review whether pain assessments were appropriately performed for the consented residents (eg, appropriate assessment depending on the cognition and communication level of the resident), whether the cause of pain was identified, whether a plan of care was established, and whether the appropriateness and effectiveness of the treatment were reviewed. Revisions to pain evaluation and management will be encouraged as needed. Outcome findings will be reviewed with the stakeholder team at 4 and 12 months.

a PAIN-CPG-EIT: Pain-Clinical Practice Guideline-Using the Evidence Integration Triangle.

b Pain Management CPG: Pain Management Clinical Practice Guideline.

Textbox 2. **Tidbit example 1. Many older adults, even those with cognitive impairment, may be hesitant to report pain for several reasons. Conversely, some individuals may overreport pain (see example below). One way to avoid overreporting or underreporting of pain is to make sure to ask about or objectively evaluate pain multiple times over the course of the day and to do so in a nonjudgmental way.**

**Reasons patients do not report pain**

- Perception of others and concern for being seen as weak and a desire to appear tough
- Embarrassed about having the pain and reporting it: not wanting to be dramatic
- Adjusting to what others expect: comments by health care providers like “feeling better today, right?”
- Underreporting of pain occurs when the individual thinks the pain is not important
- Fear of the pain reflecting something serious and fear of exposure to tests or the health care system
- Fear of being put on medications or given uncomfortable treatments
- Underreporting of pain in the hopes that it will mean getting sent home!
- Fear of the cost of any treatment for the pain
- Not wanting family to know about the pain as they do not want to upset or concern them
- Not wanting to inconvenience the health care provider: they do not want to upset their favorite nurse!
- Listening to what others say about their pain: “that should not hurt you anymore!” or “the x-ray was fine, so you should not have any pain”

**Reasons patient over report pain**

- A way to make sure that they are taken seriously and that the pain is treated
- Medication seeking for nonopioid or opioid medications
- To accelerate the time to treatment
- Concerns about discrimination and not being treated because of racial or ethnic factors
- To receive social support and caring from others
- To get the secondary gains of pain, that is, social support or getting out of going to an activity or family event
- Fear of future pain or pain exacerbation by being treated more aggressively NOW

### EO Intervention

Communities randomized to EO are provided with staff education using a PowerPoint presentation we developed for component II of the PAIN-CPG-EIT intervention. They are also given access to an online copy of the Pain Management CPG. We offer the education in the preferred format, although the communities have requested that it be delivered face-to-face.

### Measures

Community-level and resident-level descriptive data and outcomes are obtained by trained research evaluators who are blind to the treatment condition (treatment vs EO control). All resident data are obtained from medical records and direct observation at baseline and at 4 and 12 months.

Community descriptive data are obtained from the following sources: designated administrative staff for community-relevant data (eg, staffing) and Nursing Home Care Compare [46]. The following data are obtained at baseline to describe communities and consider confounders: bed size, profit or nonprofit ownership status, urban or rural, and star ratings [47]. At baseline, 4 months, and 12 months, we will also obtain staffing ratio and mix (number of nursing assistants; nurses [registered; licensed practical nurses], activity staff, primary care providers [nurse practitioners, physician assistants, and physicians], therapists [physical, occupational, and speech], social workers, pharmacists, and activities staff) per number of residents. Resident descriptive data obtained at baseline include age, race, sex, ethnicity, marital status, education, comorbidities, and cognitive status. Cognitive status is measured at baseline by evaluators using the BIMS [10]. The BIMS includes 3-item recall and orientation questions with scores ranging from 0 to 15. Prior use has provided evidence of reliability and validity [48]. Comorbidities are obtained based on chart review using the Cumulative Illness Rating Scale [49], which was designed for use with frail nursing home residents. A total of 13 organs or systems and psychiatric history are summed to obtain the total number of comorbidities.

Aim 1 measures include evidence of the assessment, diagnosis, and management of pain and the appropriate use of opioids. This is based on chart review for each participating resident, as described in Table 2. Based on review of the documentation in the electronic medical record, either an assessment is appropriately done or not, a diagnosis is documented, and a plan for pain management is incorporated into the care plan or not. Evidence of appropriate use of opioids is based on a 10-item measure as per regulatory survey requirements. Items include such things as whether a risk assessment for substance use disorder has been done; evidence that the simplest analgesic dosage schedules and least-invasive pain management modalities are being used; and evidence that the form of administration that is easiest for the resident is being used. Scores range from 0 to 10, with higher scores indicative of more appropriate use of opioids.

**Table 2.** PAIN-CPG-EIT^a^ outcome measures.

Measure	Description
Evidence of pain assessment	Based on evidence (yes/no) of whether an appropriate assessment was performed, as determined by the resident’s cognitive ability [50]. Specifically, the subjective assessment of pain in the Minimum Data Set should be used for residents with a BIMS^b^ score of 8 to 12 in our sample, and the objective assessment of pain items should be used among those with a BIMS score of less than 8 [50]. Diagnosis of pain: Based on evidence (yes/no) of a diagnosis of the underlying cause or causes of pain stated in the medical records. The most recent assessment at our baseline, and the 4- and 12-month data collection time points will be used for the chart review.
Diagnosis of pain	Based on evidence (yes/no) of a diagnosis of the underlying cause or causes of pain stated in the medical records.
Management of pain	Based on evidence (yes/no) of pain management addressed in the resident’s care plan.
Appropriate opioid use	Evidence of appropriate opioid use will be based on a checklist from the Pain Management CPG^c^ to determine whether an opioid is being used appropriately. A total of 10 items are included, and the evaluator is asked to indicate whether the recommended item has been established or is not applicable to the appropriate use of opioids. Higher scores indicate that opioid use is more likely to be appropriate. Items include such things as whether the resident has a condition that indicates that opioid use may be beneficial; whether a goal of treatment has been established; and whether a risk assessment for substance use disorder has been completed.

a PAIN-CPG-EIT: Pain-Clinical Practice Guideline-Using the Evidence Integration Triangle.

b BIMS: Brief Interview for Mental Status.

c Pain Management CPG: Pain Management Clinical Practice Guideline.

### Feasibility Measures

Feasibility and treatment fidelity data are established based on our ability to deliver the intervention as intended, evidence that the intervention was received as intended (scores on a knowledge test after education of staff), and that enactment occurred (eg, a community goal related to pain management achieved within 12 months) [51]. Specifically, delivery is based on evidence that the 4 components of the Pain Management CPG were provided as per the research nurse facilitator’s field notes, including descriptions of the stakeholder meetings (eg, number of individuals attending and focus of the meeting), number of staff exposed to education; activities completed each month with champions and other staff (eg, verbal encouragement around pain assessments, contests to reward staff for using innovative techniques for managing pain, and completion of chart review and quality assessments of whether staff were completing assessments, diagnosing, and managing pain). Receipt is based on staff demonstrating knowledge about pain based on completing the pain knowledge test, which includes 10 multiple-choice items reflective of information from the Pain Management CPG. The test is given to staff following the education session. A mean score of 80% or greater is considered evidence of sufficient knowledge. Enactment is based on the Goal Attainment Scale [52], which is completed at 12 months by the stakeholder team and research nurse facilitator. The goal, which is developed during the first stakeholder team meeting, is scored as achieved less than expected (−2); somewhat less than expected (−1); as expected (0); somewhat more than expected (1); or much more than expected (2).

### Data Analysis

All data are being entered into REDCap, which is a secure web-based application for building and managing online surveys and databases. At the end of the study period, descriptive statistics, including frequencies and percentages, measures of central tendency and dispersion, and appropriate visualization approaches (eg, box plots and spaghetti plots), will be performed on each outcome variable for residents. This will be done to ascertain distributional characteristics and ensure that the assumptions (eg, normality) associated with the planned statistical procedures are met. When necessary, transformations will be performed. All analyses will be conducted using an intent-to-treat philosophy. Baseline characteristics will be compared between intervention and control groups, and the relevant variables (eg, age) that differ by group will be included as covariates in hypothesis testing.

For aim 1, we will use generalized linear mixed models (GLMMs) to assess the effect of the intervention on the dichotomous outcomes (ie, pain assessment completed, diagnosis of pain, and plan for pain management documented). Linear mixed models (LMMs) will be used to assess the intervention effect on continuous outcomes (ie, PAINAD score and the checklist score for appropriate use of opioids). In all analyses, we will account for clustering of residents that may occur within the same community and correlations between repeated measurements of each resident. The fixed effects included in the models will be treatment group (PAIN-CPG-EIT vs control), time (baseline, 4 mo, and 12 mo), the group × time interaction term, and the aforementioned relevant covariates (eg, age). Random effects will include communities and residents. The hypotheses will be tested by evaluating the interaction term for each outcome variable. Exploratory analyses will be performed to assess model assumptions. Post–analysis diagnostic measures (eg, residuals) will be explored to assess model fit. All tests will use a 5% significance level. If there are significant differences in dropout between groups, we will identify baseline characteristics that may be related to dropout. Maximum likelihood methods will be used for primary analyses, which address noninformative dropout (missing at random [MAR]). If “informative” dropout appears possible, we will consider sensitivity analyses that involve adding these relevant baseline covariates to make the MAR assumption more plausible.

For aim 2, focused on treatment fidelity, descriptive statistics will be used to evaluate the hypotheses. Specifically, delivery of each component of PAIN-CPG-EIT will be based on whether it was delivered in each site randomized to treatment communities and whether the EO intervention was delivered in each site randomized to EO. It is anticipated that, in all 6 PAIN-CPG-EIT sites, all 4 components will be delivered as proposed (eg, 24 delivered/24 possible delivered, or 100% delivered), and for the 6 EO sites, education, or component II, will be delivered as proposed in all 6 sites (eg, 6 delivered/6 possible delivered). Receipt of the intervention will be based on posteducation testing of the staff using the pain knowledge test. Mean scores of 80% or greater in both the PAIN-CPG-EIT group and the EO group will be considered sufficient evidence of treatment fidelity based on receipt. Lastly, enactment is based on the 12-month assessment of the Goal Attainment Scale. Scores of 0 (indicative of “as expected”), 1 (“somewhat more than expected”), or 2 (“much more than expected”) provide evidence of enactment based on achieving the identified goal.

Aim 3 hypothesis (a) will be evaluated using a Rasch analysis in the Winsteps program to assess measurement invariance within an item-response theory framework and through a differential item functioning (DIF) analysis. Measurement invariance assessed using a DIF analysis focuses on evaluating the equivalence of a construct or items on a measure across groups to demonstrate that the construct or item has the same meaning to those groups or across repeated measurements. Hypothesis (b), focusing on comparisons between groups for dichotomous findings, will be analyzed using GLMMs, and comparisons between groups for continuous outcomes will be analyzed using LMMs, as described in aim 1 hypothesis testing. The fixed effects will be group (male vs female or Black vs White residents, respectively), time, and the group × time interaction, as well as potential covariates.

## Results

The study was initiated in September 2023. As delineated in Table 3, we have completed all activities (recruitment, data collection, and interventions) in cohorts 1 and 2, and recruitment has been completed at 2 of the cohort 3 sites, with treatment activities initiated. Recruitment at the third cohort 3 site has been initiated. Recruitment for the fourth cohort 3 site will begin in September 2026. The study is expected to be completed by December 2027. The communities have been extremely receptive to all study-related activities, including recruitment; data collection, by providing access to the electronic health records for participating residents; and participation in intervention-related activities. We have recruited 208 participants from the first 10 sites.

**Table 3.** Study timeline.

Study months	Calendar period	Study activities	Status
Month 1‐4: initial planning/cohort 1 setup	September 2023 to December 2023	Completion of IRB^a^ approval, Data Safety Monitoring Board meeting to approve protocol, clinical trials completion, staff training, finalizing community recruitment, and randomization. Cohort 1 coordination with communities; PAIN-CPG-EIT^b^ planning for stakeholder meeting; develop listserv of champions and stakeholders for weekly Tidbit emails. EO^c^: planning for education	Completed as per timeline
Month 5‐17: cohort 1 intervention and data collection	January 2024 to January 2025	PAIN-CPG-EIT intervention: implementation of components of PAIN-CPG-EIT; Recruitment of 25 residents per community (human participants). Ongoing community and resident data collection (baseline, 4 mo, and 12 mo). EO intervention: coordinated and provided the educational in-service; recruitment of 25 residents per community. Ongoing: community and resident data collection (baseline, 4 mo, and 12 mo)	Completed as per timeline
Month 18‐20: cohort 2 setup	February 2025 to April 2025	Cohort 2 Coordination with communities; PAIN-CPG-EIT planning for face-to-face training and identification of stakeholders and champions; develop listserv for weekly Tidbit emails	Completed as per timeline
Month 21‐33: cohort 2 intervention and data collection	May 2025 to May 2026	PAIN-CPG-EIT Intervention and EO intervention: Same process as for cohort 1. Ongoing: data collection.	Completed as per timeline
Month 34‐36: cohort 3 setup	June 2026 to August 2026	Cohort 3 coordination with communities; PAIN-CPG-EIT planning for face-to-face training	Completed as per timeline
Month 37‐49: cohort 3 intervention and data collection	June 2026 to August 2027	PAIN-CPG-EIT intervention and EO intervention: same process as for cohorts 1 and 2. Ongoing: data collection	Ongoing
Month 50-60: data analysis and manuscript development	September 2027 to June 2028	Data analysis and manuscript development	Will be completed per timeline

a IRB: Institutional Review Board.

b PAIN-CPG-EIT: Pain-Clinical Practice Guideline-Using the Evidence Integration Triangle.

c EO: education only.

## Discussion

### Principal Findings

Despite sufficient evidence noting that pain is not well assessed, diagnosed, or adequately treated among older adults living with dementia in nursing homes, there is limited use of guidelines and interventions to improve pain assessment, diagnosis, and management. As noted earlier, the state operations manual for long-term care [29], specifically F-Tag F697, requires that pain management be provided to residents who require such services, and that the management be consistent with professional standards of practice, the comprehensive person-centered care plan, and the residents’ goals and preferences. The Pain Management CPG provides the resources and tools to achieve this.

Unfortunately, there are many challenges to the implementation and use of the Pain Management CPG. Nurses may lack the skills to appropriately evaluate pain in residents, particularly those with dementia, and may assume the resident has no pain when observing him or her lying quietly in bed. It is possible that, if repositioned or transferred to a standing position, the resident might experience significant pain. The pain may only be noticed through facial grimacing or by resisting the care being provided, hitting the caregiver, or pushing him or her away.

The PAIN-CPG-EIT provides the components necessary to successfully implement the Pain Management CPG-EIT in real-world clinical settings. The PAIN-CPG-EIT approach overcomes challenges by providing staff with the appropriate tools and resources to assess, diagnose, and manage pain and helps motivate them to do so. The guidance and motivation provided by our research nurse facilitator, working with the identified unit champions, are critical to behavior change among staff and residents. Pilot testing of the implementation approach provided preliminary evidence that all 4 components of the intervention were delivered [53]. Furthermore, there was some noted improvement in the assessment approaches used by the nurses, some improvement in the use of nonpharmacologic interventions for pain, elimination of medications no longer needed for pain management, and some improvement in evaluating residents for the appropriate use of opioids. There was no evidence of improvement in care plans. Unfortunately, care plans have become computerized and generic in nature. Consequently, it is particularly critical to focus on personalizing care plans for pain and making these more patient-centered (eg, being clear on what type of nonpharmacologic approach is effective with the patient, such as performing range-of-motion exercises or taking the resident for brief walks to improve pain associated with degenerative joint disease).

### Study Strengths and Limitations

The strengths of this study protocol include the use of a strong theoretical and evidence-based approach to pain as delineated in the Pain Management CPG. Furthermore, the team has extensive research experience engaging nursing homes in research and recruiting residents living with dementia [45]. Study challenges include those well known to any work in nursing homes: staffing and turnover, engagement, lack of resources, lack of administrative support, and the tendency to focus solely on regulatory issues vs care. Our focus on pain management and implementation of the Pain Management CPG using the PAIN-CPG-EIT overcomes these issues by addressing a required regulatory care issue (F-Tag F697), including the stakeholder team in the implementation process, anticipating staff turnover by incorporating education into onboarding and making it easy to access, and incorporating the evaluation and management of pain into routine work-related activities. The findings from this study will provide evidence of the effectiveness of using the Pain Management CPG. Furthermore, this study has the potential to provide an example of how to best integrate the use of clinical practice guidelines in nursing homes.

## Supplementary material

10.2196/72985 Peer Review Report 1 Peer review report by the BMHO - Biobehavioral Medicine and Health Outcomes Study Section, Risk, Prevention and Health Behavior Integrated Review Group, National Institute of Aging (National Institutes of Health, USA).

## Abbreviations

BIMS: Brief Interview for Mental Status
CDR: Clinical Dementia Rating Scale
DIF: differential item functioning
EIT: evidence integration triangle
EO: education only
FAQ: Functional Activities Questionnaire
GLMM: generalized linear mixed model
LMM: linear mixed model
MAR: missing at random
Pain Management CPG: Pain Management Clinical Practice Guideline
PAIN-CPG-EIT: Pain-Clinical Practice Guideline-Using the Evidence Integration Triangle
PAINAD: Pain Assessment in Advanced Dementia
PALTMed: Post Acute and Long-Term Care Medical Association

## References

[1] Cole CS, Blackburn J, Carpenter JS, Chen CX, Hickman SE (2023). Pain and associated factors in nursing home residents. Pain Manag Nurs.

[2] Cole CS, Carpenter JS, Chen CX, Blackburn J, Hickman SE (2022). Prevalence and factors associated with pain in nursing home residents: a systematic review of the literature. J Am Med Dir Assoc.

[3] Achterberg W, Lautenbacher S, Husebo B, Erdal A, Herr K (2019). Pain in dementia. Pain Rep.

[4] Helvik AS, Bergh S, Tevik K (2023). A systematic review of prevalence of pain in nursing home residents with dementia. BMC Geriatr.

[5] Hoti K, Atee M, Chivers P, Vahia I, Hughes J (2023). Technology-guided assessment of vocalisations and their diagnostic value as pain indicators for people living with dementia. Age Ageing.

[6] Agit A, Balci C, Yavuz BB (2018). An iceberg phenomenon in dementia: pain. J Geriatr Psychiatry Neurol.

[7] Lichtner V, Dowding D, Esterhuizen P (2014). Pain assessment for people with dementia: a systematic review of systematic reviews of pain assessment tools. BMC Geriatr.

[8] Chow S, Chow R, Lam M (2016). Pain assessment tools for older adults with dementia in long-term care facilities: a systematic review. Neurodegener Dis Manag.

[9] Chibnall JT, Tait RC (2001). Pain assessment in cognitively impaired and unimpaired older adults: a comparison of four scales. Pain.

[10] (2025). Brief Interview for Mental Status (BIMS) calculator. The Calculator.

[11] Brennan PL, Greenbaum MA, Lemke S, Schutte KK (2019). Mental health disorder, pain, and pain treatment among long-term care residents: evidence from the Minimum Data Set 3.0. Aging Ment Health.

[12] Coutinho C, Santos S, Queirós A, Silva AG (2019). Capacity measures explain only a small part of self-reported disability in institutionalized ambulatory older adults: an observational study. Physical Occupational Therapy Geriatr.

[13] Jeon YH, Tudball J, Nelson K (2019). How do residents of aged care homes perceive physical activity and functional independence? A qualitative study. Health Soc Care Community.

[14] Rostad HM, Grov EK, Utne I, Halvorsrud L (2020). Characteristics of nursing home residents with late-stage dementia and pain [Article in Norwegian]. Sykepleien.

[15] Wróblewska I, Talarska D, Wróblewska Z, Susło R, Drobnik J (2019). Pain and symptoms of depression: international comparative study on selected factors affecting the quality of life of elderly people residing in institutions in Europe. BMC Geriatr.

[16] Wagatsuma S, Yamaguchi T, Berge LI (2021). How, why and where it hurts-breaking down pain syndrome among nursing home patients with dementia: a cross-sectional analysis of the COSMOS trial. Pain Manag Nurs.

[17] Beerens HC, Zwakhalen SMG, Verbeek H, Ruwaard D, Hamers JPH (2013). Factors associated with quality of life of people with dementia in long-term care facilities: a systematic review. Int J Nurs Stud.

[18] Corbett A, Husebo B, Malcangio M (2012). Assessment and treatment of pain in people with dementia. Nat Rev Neurol.

[19] Corbett A, Nunez KM, Smeaton E (2016). The landscape of pain management in people with dementia living in care homes: a mixed methods study. Int J Geriatr Psychiatry.

[20] van Kooten J, van der Wouden JC, Sikkes SAM, Smalbrugge M, Hertogh C, Stek ML (2017). Pain, neuropsychiatric symptoms, and quality of life of nursing home residents with advanced dementia in the Netherlands: a cross-sectional study. Alzheimer Dis Assoc Disord.

[21] Torvik K, Kaasa S, Kirkevold O, Rustøen T (2010). Pain and quality of life among residents of Norwegian nursing homes. Pain Manag Nurs.

[22] Boltz M, Resnick B, Kuzmik A (2021). Pain incidence, treatment, and associated symptoms in hospitalized persons with dementia. Pain Manag Nurs.

[23] Kaiho Y, Sugawara Y, Sugiyama K (2017). Impact of pain on incident risk of disability in elderly Japanese: cause-specific analysis. Anesthesiology.

[24] Kolanowski A, Mogle J, Fick DM (2015). Pain, delirium, and physical function in skilled nursing home patients with dementia. J Am Med Dir Assoc.

[25] Habiger TF, Achterberg WP, Flo-Groeneboom E, Mannseth J, Husebo BS (2021). Managing pain and psychosis symptoms in nursing home patients: results from a cluster-randomized controlled trial (COSMOS). J Am Med Dir Assoc.

[26] van de Beek SH, Erdal A, Husebø BS, Vislapuu M, Achterberg WP, Caljouw MAA (2024). Impact of pain and neuropsychiatric symptoms on activities in nursing home residents (COSMOS Trial). J Am Med Dir Assoc.

[27] Hunt LJ, Ritchie CS, Cataldo JK, Patel K, Stephens CE, Smith AK (2018). Pain and emergency department use in the last month of life among older adults with dementia. J Pain Symptom Manage.

[28] Robitaille A, Adams M, Heckman G (2024). Pain in Canadian long-term care homes: a call for action. J Am Med Dir Assoc.

[29] (2021). State operations manual appendix PP—guidance to surveyors for long term care facilities. https://www.cms.gov/Regulations-and-Guidance/Guidance/Manuals/Downloads/som107ap_pp_guidelines_ltcf.pdf.

[30] Levenson S, Resnick B (2021). The AMDA pain CPG: a different kind of trustworthy clinical guideline. J Am Med Dir Assoc.

[31] Gregson J, Foerster SB, Orr R (2001). System, environmental, and policy changes: using the social-ecological model as a framework for evaluating nutrition education and social marketing programs with low-income audiences. J Nutr Educ.

[32] Bandura A (1995). Self-Efficacy in Changing Societies.

[33] Bandura A (1997). Self-Efficacy: The Exercise of Control.

[34] Bandura A (2004). Health promotion by social cognitive means. Health Educ Behav.

[35] Suresh K (2011). An overview of randomization techniques: an unbiased assessment of outcome in clinical research. J Hum Reprod Sci.

[36] Boltz M, Chippendale T, Resnick B, Galvin JE (2015). Testing family-centered, function-focused care in hospitalized persons with dementia. Neurodegener Dis Manag.

[37] Mansbach WE, Mace RA, Clark KM (2014). Differentiating levels of cognitive functioning: a comparison of the Brief Interview for Mental Status (BIMS) and the Brief Cognitive Assessment Tool (BCAT) in a nursing home sample. Aging Ment Health.

[38] Galvin JE, Roe CM, Powlishta KK (2005). The AD8: a brief informant interview to detect dementia. Neurology.

[39] Galvin JE, Roe CM, Xiong C, Morris JC (2006). Validity and reliability of the AD8 informant interview in dementia. Neurology.

[40] O’Bryant SE, Waring SC, Cullum MC (2008). Staging dementia using Clinical Dementia Rating Scale Sum of Boxes scores: a Texas Alzheimer’s research consortium study. Arch Neurol.

[41] Pfeffer RI, Kurosaki TT, Harrah Jr CH, Chance JM, Filos S (1982). Measurement of functional activities in older adults in the community. J Gerontol.

[42] Pfeffer RI, Kurosaki TT, Harrah Jr CH, Chance JM, Filos S (1982). Functional Activities Questionnaire. J Gerontol.

[43] Resnick B, Boltz M, Galik E, Ellis J, Kuzmik A, Drazich B (2023). Optimal approach for assessing evidence of dementia for research studies. West J Nurs Res.

[44] Resnick B, Gruber-Baldini AL, Zimmerman S (2009). Nursing home resident outcomes from the Res-Care intervention. J Am Geriatr Soc.

[45] Resnick B, Van Haitsma K, Kolanowski A (2021). Implementation of the Evidence Integration Triangle for behavioral and psychological symptoms of dementia (EIT-4-BPSD) in care communities. Nurs Outlook.

[46] Centers for Medicare and Medicaid Services (2022). Nursing home care compare. Medicare.gov.

[47] (2021). Quality of resident care. The Centers for Medicare & Medicaid Services.

[48] Saliba D, Buchanan J (2012). Making the investment count: revision of the Minimum Data Set for nursing homes, MDS 3.0. J Am Med Dir Assoc.

[49] Linn BS, Linn MW, Gurel L (1968). Cumulative illness rating scale. J Am Geriatr Soc.

[50] Kaasalainen S, Crook J (2004). An exploration of seniors’ ability to report pain. Clin Nurs Res.

[51] Bellg AJ, Borrelli B, Resnick B (2004). Enhancing treatment fidelity in health behavior change studies: best practices and recommendations from the NIH Behavior Change Consortium. Health Psychol.

[52] Kiresuk T, Smith A, Cardillo J (1994). Goal Attainment Scaling: Applications, Theory and Measurement.

[53] Resnick B, McPherson R, Galik E (2024). Pilot testing implementation of the pain management clinical practice guideline in nursing homes. Geriatr Nurs.

