# Supplementary material for: Testing the Implementation of the PAIN-CPG-EIT: Protocol for a Randomized Clinical Trial
Source: JMIR Res Protoc. 2026 Sep 30;15:e72985. doi: 10.2196/72985 (PMC13626648; doi:10.2196/72985)
Supplement: Supplementary file 1 [file resprot-v15-e72985-s001.pdf]

SUMMARY STATEMENT
(Privileged Communication)

PROGRAM CONTACT:
Barbara Radziszewska
301-435-3046
radziszb@mail.nih.gov

Release Date: **03/06/2023**
Revised Date:

Application Number: **1 R01 AG082676-01**

Principal Investigator

RESNICK, BARBARA

Applicant Organization: UNIVERSITY OF MARYLAND BALTIMORE

Review Group: **BMHO**
Biobehavioral Medicine and Health Outcomes Study Section

Meeting Date: **02/06/2023**
Council: **MAY 2023**
Requested Start: **07/01/2023**

RFA/PA: **PA20-183**
PCC: **4CCTSBR**
Dual IC(s): **NR**

Project Title: **Testing the Pain-CPG-EIT**

SRG Action: **Impact Score:25 Percentile:13**
Next Steps: **Visit https://grants.nih.gov/grants/next_steps.htm**
Human Subjects: 48-At time of award, restrictions will apply
Animal Subjects: 10-No live vertebrate animals involved for competing appl.
Gender: 1A-Both genders, scientifically acceptable
Minority: 1A-Minorities and non-minorities, scientifically acceptable
Age: 8A-Only Older Adults, scientifically acceptable

Project Year	Direct Costs Requested	Estimated Total Cost
1	328,377	507,343
2	328,377	507,343
3	328,377	507,343
4	328,377	507,343
5	328,377	507,343
TOTAL	1,641,885	2,536,715

ADMINISTRATIVE BUDGET NOTE: The budget shown is the requested budget and has not been adjusted to reflect any recommendations made by reviewers. If an award is planned, the costs will be calculated by Institute grants management staff based on the recommendations outlined below in the COMMITTEE BUDGET RECOMMENDATIONS section.

1R01AG082676-01 RESNICK, BARBARA

PROTECTION OF HUMAN SUBJECTS UNACCEPTABLE

RESUME AND SUMMARY OF DISCUSSION: This application proposes a cluster RCT to evaluate the efficacy and treatment fidelity of an intervention to improve assessment, diagnosis and management of pain, and decrease pain intensity in older adult dementia patients in nursing homes. During discussion, the panel agreed the significance of enhancing newly created pain management clinical practice guidelines (CPG) for implementation by testing an existing pain intervention for older patients with dementia is high, with potential to advance clinical care and improve functioning and quality of life in the target population. Reviewers noted using stakeholder involvement to adapt the guidelines to each community's needs and examining race and sex differences in pain care improvement by this outstanding PI and strong investigative team are innovative. Additional strengths identified include strong preliminary data supporting feasibility, a well-articulated theoretical foundation, a rigorous methodology with an appropriate study design and comparison arm, a thorough description of the intervention, use of the evidence integration triangle (EIT) to define the implementation process and address facilitators/barriers, and prospective assessment 12 months post-implementation. Reviewers also identified addressable weaknesses such as no expertise in implementation science on the team and lack of clarity on the integration of Aim 3 with the first two aims. Overall, the panel agreed the application's strengths outweighed its weaknesses and will have a moderate impact on advancing clinical care of pain management for older dementia patients in nursing homes.

DESCRIPTION (provided by applicant): Pain is experienced by 30% to 80% of residents living with dementia in nursing homes. For those with moderate to severe dementia verbal reporting of pain may not be reliable and observational approaches are recommended as pain in these individuals is more likely to present with facial expressions or behaviors such as aggression, agitation, or restlessness. Lack of accurate pain assessment results in untreated or over- treated pain. Untreated pain can lower quality of life, negatively impact function, impair sleep, increase depression, agitation, aggression, resistiveness to care and use of psychotropic medications. Further, the evaluation, management and treatment of pain are complicated by differences in pain sensitivity, verbal reporting or presentation of pain between genders, races and ethnicities. Although inconsistent, in some studies individuals living with dementia who were Black were more likely to have depression and sleep disturbances associated with pain and less likely to be treated for pain when compared to White residents. There are evidence based processes for assessment and management of pain using pharmacologic and nonpharmacological approaches. These were reviewed and included within the Pain Management Clinical Practice Guideline (Pain Management CPG) recently developed by AMDA: The Society for Post-Acute and Long-Term Care Medicine. There are, however, many challenges to translating the use of Clinical Practice Guidelines into clinical settings. To overcome these challenges we developed and previously tested a theoretically based approach and merged this approach with the Pain Management CPG, which is referred to as the PAIN-CPG-EIT. The PAIN-CPG-EIT involves a research nurse facilitator working with an identified community champion(s) and stakeholder team for 12 months to provide the following four components: Component I: Establishing and meeting monthly with a Stakeholder Team; Component II: Education of the staff; Component III: Mentoring and motivating the staff to address pain; Component IV: Ongoing evaluation of resident pain outcomes. Twelve communities will be included with 25 residents living with dementia and pain recruited from each community. Six communities will be randomized to treatment (PAIN-CPG-EIT) and six randomized to education only (EO) which involves providing the same education to staff as is done in Component II of PAIN-CPG-EIT. The primary aim of this study is to test the effectiveness of use of the PAIN- CPG-EIT to improve the assessment, diagnosis and management of pain and decrease pain intensity among nursing home residents living with dementia between baseline, 4 and 12 months and evaluate

treatment fidelity. A secondary aim of the study is to consider differences in measurement, treatment and response to treatment between male and female and Black versus White residents living with dementia. Findings from this study will help build on the currently limited information about pain presentation and management among older adults living with dementia in nursing homes and improve health equity of aging populations experiencing pain.

PUBLIC HEALTH RELEVANCE: The purpose of this study is to improve the assessment, diagnosis, and management of pain among residents living with dementia in nursing homes by testing the use of the new Pain Management Clinical Practice Guideline and compare differences in assessment, diagnosis and treatment of pain among male and female and Black versus White residents living with dementia.

CRITIQUE 1

Significance: 1
Investigator(s): 2
Innovation: 3
Approach: 3
Environment: 1

Overall Impact: This R01 application proposes to evaluate the effectiveness of the implementation of a set of recently developed Pain Management Clinical Practice Guideline among patients with dementia in community nursing home facilities. The outstanding PI and Co-Investigators have a strong track record and experience executing similar studies for other elder care indications, and seek to apply that knowledge base to pain management. There is a major need for such an intervention, given the complexities of pain assessment and treatment in patients with dementia in nursing homes. The approach has several strengths, including a strong theoretical basis, a clear plan for implementation, and appropriate clinical trial design decisions, such as cluster randomization of nursing homes rather than individual residents. The biggest drawback of this application is the lack of clarity and integration of Aim 3 with the other Aims. Additionally, relatively little quantitative preliminary data is presented here in support of the grant, despite the fact that this team has done similar work in the past and published extensively; the limited elaboration on quantitative data gathered from prior work raises some concern regarding the rigor of prior research. Overall, however, these weaknesses do not outweigh the substantial strengths, as this study is viewed to have a potential for significant impact.

1. Significance:

Strengths

- The process of establishing protocols for proper pain assessments and runways for the development and implementation of pain treatment plans is inconsistently applied across health system settings, and appears to be particularly deficient in the context of dementia care. A rigorous program that can be disseminated across different dementia care settings would have substantial value and societal impact.
- By engaging both providers and patients, the proposed program increases the chances of success, relative to one that focus on one group or the other.
- Engagement of a wide range of stakeholders increases the generalizability of the findings.
- Explicit focus on racial and ethnic pain disparities is woven into the fabric of the study.

Weaknesses

- None noted.

2. Investigator(s):

Strengths

- The PI Dr. Resnick is a nursing science leader with an exceptional track record of scholarship, strong pain-related research track record in older adults, and prior experience applying the methods proposed here in different contexts (e.g., dementia care).
- Co-I Galik is also highly accomplished and has a long history of working with Dr. Resnick.

Weaknesses

- A substantial amount of outcome data comes from EMR chart review, but there is not an EMR medical informatics expert on the study team.

3. Innovation:

Strengths

- Titrating the program to the needs and strengths of each community participant organization ensures that program structure can be dynamic and respond to cultural or socioeconomic needs of different communities.

Weaknesses

- The project lacks innovation related to harnessing the electronic medical record.

4. Approach:

Strengths

- The approach is built on a strong and well-articulated theoretical premise with a foundation in a technique (EIT) that is evidence-based.
- Cluster-randomized approach to study residents within different nursing home communities is a strong design choice to control for heterogeneity between sites.
- The team has already generated support from 12 nursing homes for participation in this study.
- The description of the intervention and plans for execution are thorough and lend confidence that the team can successfully execute the plan.

Weaknesses

- Although several references to prior work were cited, very little quantitative preliminary data is presented in support of this application. One of their prior cited publications demonstrated evidence for successful implementation of a similar approach for behavioral and psychological distress, but did not find evidence for effectiveness. Thus, there is a concern regarding the rigor of prior research in relation to the proposed study that is not sufficiently addressed because preliminary data are not provided in sufficient detail and prior treatment failures not discussed.
- The framing and rationale for Aim 3 are not clear, and it is not well integrated with the other Aims. It is not clearly explained why, for example, whether variance/invariance in the PAINAD would be expected to change after the intervention, or why the focus is on invariance in the first place. Greater clarity and integration of this aim with the others is needed.

5. Environment:

Strengths

- The University of Maryland School of Nursing has all of the necessary research, computing, and administrative supports to facilitate a successful program.
- The team has a track record of engaging with community nursing home facilities.

Weaknesses

- None noted.

Study Timeline:

Strengths

- The study is broken into 3 cohort enrollment and intervention phases, which provides a useful structure to support feasibility.

Weaknesses

- None noted.

Protections for Human Subjects:

Acceptable Risks and/or Adequate Protections

- Acceptable.

Data and Safety Monitoring Plan (Applicable for Clinical Trials Only):

Unacceptable

- The DSMP is generally light on details and will need to be expanded prior to study start.

Inclusion Plans:

- Sex/Gender: Distribution justified scientifically
- Race/Ethnicity: Distribution justified scientifically
- Inclusion/Exclusion Based on Age: Distribution justified scientifically
- All acceptable.

Vertebrate Animals:

Not Applicable (No Vertebrate Animals)

- N/A

Biohazards:

Not Applicable (No Biohazards)

- N/A

Resource Sharing Plans:

Acceptable

- Acceptable

Budget and Period of Support:

Recommend as Requested

- Acceptable.

CRITIQUE 2

Significance: 2
Investigator(s): 1
Innovation: 2
Approach: 3
Environment: 1

Overall Impact: The overall aim of the project is to assess the effect of PAIN-CPG-EIT, a pain management clinical practice guideline plus a nurse facilitator who works with a community champion and stakeholder team to address pain assessment and management in residents living with dementia in nursing homes compared to an education only group on 12 residential communities (6 randomized to each group) with 25 residents in each community. A secondary focus of the project is to examine differences in assessment and management of pain among male/female and Black/White residents with dementia. Significance of the project is high given prevalence of pain in older adults living with dementia in nursing homes as well as significant challenges to pain assessment/management in this population. The investigatory team is outstanding and the environment is strong and supports the proposed project. Innovation is supported by the implementation of a systematic, evidence-based, and theoretically informed approach to pain assessment and management in older adults living with dementia in nursing homes that includes stakeholder and community engagement. The approach has many strengths, including preliminary evidence of high rigor to support the study aims, project feasibility and study design, and an approach that addresses significant gaps in the literature. Weaknesses are present but minor. Overall this project is deemed as having high potential for clinical and scientific impact.

1. Significance:

Strengths

- Pain is prevalent in older adults in nursing home settings and extremely challenging to assess in patients living with dementia. When pain is undertreated in older adults, there are significant and negative impacts on functioning and quality of life. Verbal pain assessment in older adults with dementia may not be possible, underscoring the need for observational approaches.
- Evidence supports racial/ethnic disparities in pain assessment and management among older, Black and male adults living with dementia, thus the focus on racial disparities lends to study significance.
- The proposed intervention relies on functional assessments, which are highly relevant to pain, as well as non-pharmacological interventions, which are highly relevant to older adults and can mitigate opioid-related harm.

Weaknesses

- Strategies to address racial/ethnic disparities in pain assessment/management are not incorporated into the intervention, which weakens significance.

2. Investigator(s):

Strengths

- The PI, Dr. Resnick, has a PhD in research and undergraduate and graduate training in nursing/gerontological nursing. Dr. Resnick is a Professor of Nursing with expertise in care of older adults with dementia and pain with a strong track record of peer-reviewed research and extramural funding and has the relevant expertise and experience to execute the proposed study aims.
- Co-investigator Dr. Galik has a doctoral in nursing research and is trained as a gerontological nurse with substantial expertise in working with older adults with dementia and neuropsychiatric symptoms. Dr. Galik has a strong track record of scholarship and is well-poised to serve as co-investigator on the proposed study.
- Dr. Zhu has a PhD in statistics and has a significant collaborative history with Dr. Resnick.
- Overall the investigatory team is strong and has the necessary expertise to carry out the proposed study.

Weaknesses

- None noted.

3. Innovation:

Strengths

- The implementation of a systematic, evidence-based, and theoretically informed approach to pain assessment and management in older adults living with dementia in nursing homes is novel and innovative.
- The multiple components of the EIT, including stakeholder and champion involvement is innovative. The flexibility of allowing communities to identify unique barriers/facilitators will lend to relevance and sustainability of the intervention.

Weaknesses

- Diversifying stakeholder involvement, including input from family members/caregivers, particularly in the context of racial/ethnic and other social determinants of help could add important input to ultimately develop culturally adapted approaches to the intervention, which is an important oversight.

4. Approach:

Strengths

- The approach is guided by evidence, and strong conceptual and theoretical frameworks.
- Integration of the Pain Management CPG (guidelines) with the Evidence Integration Triangle, which aims to decrease behavioral symptoms associated with dementia and increase physical activity, is an improvement over current methods of pain assessment/management in this area. In particular, the inclusion of stakeholder and community engagement lends to a relevant and sustainable intervention.

- The EIT also addresses barriers to pain assessment/management in the proposed populations/settings and is created in a way that it can be implemented feasibly and reliably in multiple settings.
- The engagement of stakeholders lend to the sustainability of the intervention over time.
- Preliminary data of high rigor support the aims, feasibility, and premise of the proposed project.
- The use of cluster randomization is well-justified to prevent treatment contamination and power analysis supports the proposed sample size. Feasibility of participant recruitment is supported.
- Letters of support demonstrate feasibility of enrolling the 12 proposed nursing home sites.
- The intervention components are well described, logical, and including team establishment, education, mentoring and motivating, as well as ongoing evaluation which appears comprehensive and will lend to establishing an evidence-base for the intervention.
- Treatment fidelity is well described.

Weaknesses

- The intervention approach involves many different stakeholder members at each nursing home, which may make consistent inclusion of each different member across all sites challenging and there are no feasibility data to support successful inclusion of all members across sites.
- The study is framed, in significant part, as addressing pain disparities among Black male adults, but other than descriptive data and analysis of outcomes by race/age there is no actual proposed means of addressing disparities by the intervention, which is a significant weakness. It would be helpful to identify barriers/facilitators to racial disparities in pain assessment/management in this population and incorporate strategies to address disparities in the intervention or not frame the intervention as relevant for racial disparities.
- It is not clear how variations in nursing home characteristics may be adjusted for in the analytic approach.
- It may be helpful to include patients' family members/caregivers into the assessment strategy, particularly with the goal to address racial/ethnic pain disparities.

5. Environment:

Strengths

- The University of Maryland, Baltimore has strong research and clinical infrastructure to support the proposed project.

Weaknesses

- None noted.

Study Timeline:

Strengths

- Detailed and supports completion of study aims within the proposed timeline.

Weaknesses

- None noted.

Protections for Human Subjects:

Acceptable Risks and/or Adequate Protections

- Minimal risk study that will be conducted with personnel with expertise in working with older adults. Adequate protections from risk detailed.

Data and Safety Monitoring Plan (Applicable for Clinical Trials Only):

Acceptable

- Sufficient DSMP described with appropriate team members and activities.

Inclusion Plans:

- Sex/Gender: Distribution justified scientifically
- Race/Ethnicity: Distribution justified scientifically
- Inclusion/Exclusion Based on Age: Distribution justified scientifically
- Participants in the proposed project will include adults ages 65 years and older living with dementia in nursing homes. Based upon demographic data of residents in nursing homes in the geographic area, it is expected that 70% of participants will be female and 65% will be white and 30% will be Black.

Vertebrate Animals:

Not Applicable (No Vertebrate Animals)

Biohazards:

Not Applicable (No Biohazards)

Resource Sharing Plans:

Acceptable

Budget and Period of Support:

Recommend as Requested

CRITIQUE 3

Significance: 3
Investigator(s): 1
Innovation: 1
Approach: 2
Environment: 1

Overall Impact: This R01 application proposes an RCT to evaluate the efficacy of a clinical practice guideline-based intervention to improve the assessment and management of pain in dementia patients in the nursing home setting. Pain is common in patients with dementia living in nursing homes but optimal assessment and management of pain in these individuals is challenging. New clinical practice guidelines (CPG) for assessing and managing pain in nursing home setting have been released but

have not yet been implemented widely. The project's plans to use the investigators' previously tested approach to enhance guideline implementation and improve pain assessment and management (PAIN-CPG-EIT) in dementia patients has high clinical significance. The nurse-led intervention is grounded in the social ecological model and social cognitive theory which is a strength. The intervention's focus on changing nursing home staff behavior to effect improved patient care via CPG implementation also enhances significance, and plans to examine race and sex differences in effects of the intervention add a degree of scientific significance to the project. The rigor of prior research cited to support the project is high overall. However, the CPG referenced appears to be targeting nursing home residents in general rather than dementia patients specifically. Discussion of using non-pharmacological approaches (which require active patient participation) as first line treatments as part of the CPG does not address the likely barriers to use of many such interventions in individuals with moderate to severe dementia – this could reduce significance of the proposed CPG-based intervention in clinical practice to some degree. A rationale for Aim 3 hypotheses regarding sex differences is also not provided. The project is supported by the investigators' preliminary work using this approach to improve behavioral symptoms and activity in dementia patients. Although the investigators' prior work evaluating implementation of 2004 pain CPG guidelines in nursing homes supports the feasibility of the project, the fact that the prior (simpler) CPG-focused intervention did not significantly alter pain outcomes raises some questions as to whether planned implementation of newer CPG-based pain management approaches will improve management of dementia patients' pain in a meaningful way. That is, does inadequate dementia pain management result from suboptimal implementation of CPGs or is the issue that the CPGs do not improve pain even when implemented optimally? This latter important question is not explicitly addressed in the aims and analyses although data to be collected appears suitable for addressing this issue. The PI is an experienced nurse researcher with expertise related to enhancing care of dementia patients in the nursing home setting. She has conducted previous research on implementation of an earlier pain CPG (2004). Other proposed team members add further expertise regarding dementia and pain, as well as biostatistics, and the team overall appears well-qualified to carry out the project. The approach used for enhancing integration of CPG-based pain management in dementia patients in the nursing home setting is innovative. The intervention itself appears to be incrementally novel, building on the investigators' prior work using a similar approach to address non-pain issues in dementia patients. Evaluation of race and sex differences in improvements in pain care using the proposed approach is also novel. The approach is rigorous, including an appropriate cluster-randomized clinical trial design, a pain education comparison condition, prospective assessment out to 12 months post-implementation, a well-described intervention, validated screening and pain measures, and blinded outcome assessors. One potential weakness in plans to address intervention fidelity (Aim 2) is that the goals and criteria for determining whether the CPG approach was properly implemented (Enactment) appear to be generated separately by study personnel at each nursing home site (making comparisons across sites more challenging), and it is not clear that these criteria are reviewed for suitability or consistency across sites prior to the study. Related to comments above, the analytic plan might also benefit from consideration of the question of whether potential failure to observe significant pain-reducing effects of the intervention (based on rated pain) is due to implementation issues or lack of efficacy of the CPG-based pain management approach. The research environment at University of Maryland – Baltimore appears to be strong and likely to support successful completion of the project.

Study Timeline:

Strengths

- The proposed timeline appears feasible given the investigators' past work using a similar approach.

Weaknesses

- None noted.

Protections for Human Subjects:

Acceptable Risks and/or Adequate Protections

- Plans to insure the safety and confidentiality of participants are adequate.

Data and Safety Monitoring Plan (Applicable for Clinical Trials Only):

Acceptable

- Although the study is low risk, a formal data safety monitoring committee is planned.

Inclusion Plans:

- Sex/Gender: Distribution justified scientifically
- Race/Ethnicity: Distribution justified scientifically
- Inclusion/Exclusion Based on Age: Distribution justified scientifically
- Males and females age 65 or older will be eligible and this is acceptable given the aims of the project. The race/ethnicity of the final sample is expected to parallel the local nursing home population which is acceptable.

Vertebrate Animals:

Not Applicable (No Vertebrate Animals)

Biohazards:

Not Applicable (No Biohazards)

Resource Sharing Plans:

Not Applicable (No Relevant Resources)

Budget and Period of Support:

Recommend as Requested

THE FOLLOWING SECTIONS WERE PREPARED BY THE SCIENTIFIC REVIEW OFFICER TO SUMMARIZE THE OUTCOME OF DISCUSSIONS OF THE REVIEW COMMITTEE, OR REVIEWERS' WRITTEN CRITIQUES, ON THE FOLLOWING ISSUES:

PROTECTION OF HUMAN SUBJECTS: UNACCEPTABLE

The panel agreed the Data Safety Monitoring plan was unacceptable due to limited details and needs to be expanded prior to start of the study.

INCLUSION OF WOMEN PLAN: ACCEPTABLE

INCLUSION OF MINORITIES PLAN: ACCEPTABLE

INCLUSION ACROSS THE LIFESPAN: ACCEPTABLE

COMMITTEE BUDGET RECOMMENDATIONS: The budget was recommended as requested.

Footnotes for 1 R01 AG082676-01; PI Name: RESNICK, BARBARA

NIH has modified its policy regarding the receipt of resubmissions (amended applications).See Guide Notice NOT-OD-18-197 at https://grants.nih.gov/grants/guide/notice-files/NOT-OD-18-197.html. The impact/priority score is calculated after discussion of an application by averaging the overall scores (1-9) given by all voting reviewers on the committee and multiplying by 10. The criterion scores are submitted prior to the meeting by the individual reviewers assigned to an application, and are not discussed specifically at the review meeting or calculated into the overall impact score. Some applications also receive a percentile ranking. For details on the review process, see http://grants.nih.gov/grants/peer_review_process.htm#scoring.

MEETING ROSTER

Biobehavioral Medicine and Health Outcomes Study Section
Risk, Prevention and Health Behavior Integrated Review Group
CENTER FOR SCIENTIFIC REVIEW
BMHO
02/06/2023 - 02/07/2023

Notice of NIH Policy to All Applicants: Meeting rosters are provided for information purposes only. Applicant investigators and institutional officials must not communicate directly with study section members about an application before or after the review. Failure to observe this policy will create a serious breach of integrity in the peer review process, and may lead to actions outlined in NOT-OD-22-044 at https://grants.nih.gov/grants/guide/notice-files/NOT-OD-22-044.html, including removal of the application from immediate review.

CHAIRPERSON(S)

BRUEHL, STEPHEN, PHD
PROFESSOR
DEPARTMENT OF ANESTHESIOLOGY
SCHOOL OF MEDICINE
VANDERBILT UNIVERSITY MEDICAL CENTER
NASHVILLE, TN 37212

MEMBERS

BUTLER, ASHLEY M, PHD
ASSOCIATE PROFESSOR
DEPARTMENT OF PEDIATRIC PSYCHOLOGY
BAYLOR COLLEGE OF MEDICINE
HOUSTON, TX 77030

CHOW, ERIC JESSEN, MD, MPH
ASSOCIATE PROFESSOR
PUBLIC HEALTH SCIENCES DIVISION
DEPARTMENT OF PEDIATRICS
FRED HUTCHINSON CANCER RESEARCH CENTER
SEATTLE, WA 98109

FINAN, PATRICK, PHD
PROFESSOR
DEPARTMENT OF ANESTHESIOLOGY
UNIVERSITY OF VIRGINIA SCHOOL OF MEDICINE
CHARLOTTESVILLE, VA 22904

FORTIER, MICHELLE, PHD
ASSOCIATE PROFESSOR
SUE AND BILL GROSS SCHOOL OF NURSING
UNIVERSITY OF CALIFORNIA, IRVINE
ORANGE, CA 92868

GALLO, LINDA C, PHD
PROFESSOR
DEPARTMENT OF PSYCHOLOGY
SAN DIEGO STATE UNIVERSITY
SAN DIEGO, CA 92123

GARY, REBECCA A, PHD *
PROFESSOR
NELL HODGSON WOODRUFF SCHOOL OF NURSING
EMORY UNIVERSITY
ATLANTA, GA 30322

GOLDMAN, MYLA DENISE, MD *
PROFESSOR
DEPARTMENT OF NEUROLOGY
VIRGINIA COMMONWEALTH UNIVERSITY
SCHOOL OF MEDICINE
RICHMOND, VA 23292

GRABOYES, EVAN MICHAEL, MD, MPH *
ASSOCIATE PROFESSOR
DEPARTMENT OF OTOLARYNGOLOGY
HEAD AND NECK SURGERY
MEDICAL UNIVERSITY OF SOUTH CAROLINA
CHARLESTON, SC 29425

GRIGGS, STEPHANIE ALISHA, PHD *
ASSISTANT PROFESSOR
FRANCES PAYNE BOLTON SCHOOL OF NURSING
CASE WESTERN RESERVE UNIVERSITY
CLEVELAND, OH 44106

HASSETT, AFTON L, PSYD
ASSOCIATE PROFESSOR
DEPARTMENT OF ANESTHESIOLOGY
CHRONIC PAIN AND FATIGUE RESEARCH CENTER
UNIVERSITY OF MICHIGAN MEDICAL SCHOOL
ANN ARBOR, MI 48106

IRWIN, SCOTT A., PHD, MD *
PROFESSOR
PSYCHIATRY AND BEHAVIORAL NEUROSCIENCES
DEPARTMENT
CEDARS SINAI
LOS ANGELES, CA 90048

KRONISH, IAN M, MD
ASSOCIATE PROFESSOR
DEPARTMENT OF MEDICINE
COLUMBIA UNIVERSITY IRVING MEDICAL CENTER
NEW YORK, NY 10032

MARQUEZ, DAVID X, PHD
PROFESSOR
DEPARTMENT OF KINESIOLOGY AND NUTRITION
UNIVERSITY OF ILLINOIS AT CHICAGO
CHICAGO, IL 60612

MCELFISH, PEARL, PHD *
DIRECTOR
OFFICE OF COMMUNITY HEALTH AND RESEARCH
COLLEGE OF MEDICINE
UNIVERSITY OF ARKANSAS FOR MEDICAL SCIENCES
FAYETTEVILLE, AR 72703

MEYER, JACOB D, PHD *
ASSISTANT PROFESSOR
DEPARTMENT OF KINESIOLOGY
IOWA STATE UNIVERSITY
AMES, IA 50011

MOSSAVAR-RAHMANI, YASMIN, PHD *
PROFESSOR
DIVISION OF HEALTH BEHAVIOR RESEARCH &
IMPLEMENTATION SCIENCE
DEPARTMENT OF EPIDEMIOLOGY AND POPULATION
HEALTH
ALBERT EINSTEIN COLLEGE OF MEDICINE
BRONX, NY 10461

NAPADOW, VITALY, PHD
PROFESSOR
SPAULDING REHABILITATION HOSPITAL
HARVARD MEDICAL SCHOOL
CHARLESTOWN, MA 02129

PULCINI, CHRISTIAN DONALD, MD, MPH *
ASSISTANT PROFESSOR
DIVISION OF EMERGENCY MEDICINE
DEPARTMENT OF SURGERY
COLLEGE OF MEDICINE
UNIVERSITY OF VERMONT
BURLINGTON, VT 05401

PYATAK, ELIZABETH F, PHD
ASSOCIATE PROFESSOR
DIVISION OF OCCUPATIONAL SCIENCE
AND OCCUPATIONAL THERAPY
UNIVERSITY OF SOUTHERN CALIFORNIA
LOS ANGELES, CA 90089

RENN, CYNTHIA L, PHD *
ASSOCIATE PROFESSOR
SCHOOL OF NURSING
UNIVERSITY OF MARYLAND
BALTIMORE, MD 21201

RINI, CHRISTINE, PHD
PROFESSOR
DEPARTMENT OF MEDICAL SOCIAL SCIENCES
FEINBERG SCHOOL OF MEDICINE
NORTHWESTERN UNIVERSITY
CHICAGO, IL 60611

ROPER, JENEVIEVE LYNN, PHD *
ASSISTANT PROFESSOR
DEPARTMENT OF HEALTH AND HUMAN SCIENCES
LOYOLA MARYMOUNT UNIVERSITY
LOS ANGELES, CA 90045

RUDROFF, THORSTEN, PHD *
ASSOCIATE PROFESSOR
DEPARTMENT OF HEALTH AND HUMAN PHYSIOLOGY
COLLEGE OF LIBERAL ARTS AND SCIENCES
IOWA STATE UNIVERSITY
IOWA CITY, USA, IA 52240

SCHENKER, YAEL, MD
PROFESSOR
SECTION OF PALLIATIVE CARE AND MEDICAL ETHICS
DIVISION OF GENERAL INTERNAL MEDICINE
UNIVERSITY OF PITTSBURGH
PITTSBURGH, PA 15213

SMITH, PATRICK J, PHD
ASSOCIATE PROFESSOR
DEPARTMENT OF PSYCHIATRY
SCHOOL OF MEDICINE
UNIVERSITY OF NORTH CAROLINA AT CHAPEL HILL
CHAPEL HILL, NC 27599

TAPIA, IGNACIO ESTEBAN, MD
ASSOCIATE PROFESSOR
DEPARTMENT OF PEDIATRICS
PERELMAN SCHOOL OF MEDICINE
UNIVERSITY OF PENNSYLVANIA
PHILADELPHIA, PA 19104

TEMPLE, JENNIFER L, PHD
PROFESSOR
DEPARTMENT OF EXERCISE AND NUTRITION SCIENCES
SCHOOL OF PUBLIC HEALTH AND HEALTH PROFESSIONS
UNIVERSITY AT BUFFALO
BUFFALO, NY 14214

UEBELACKER, LISA A, PHD
PROFESSOR
DEPARTMENT OF PSYCHIATRY AND HUMAN BEHAVIOR
BUTLER HOSPITAL
BROWN UNIVERSITY
PROVIDENCE, RI 02906

WEN, KUANG-YI, PHD
ASSOCIATE PROFESSOR
DEPARTMENT OF MEDICAL ONCOLOGY
THOMAS JEFFERSON UNIVERSITY
PHILADELPHIA, PA 19107

YAGGI, HENRY KLAR, MD, MPH *
PROFESSOR
SCHOOL OF PUBLIC HEALTH
YALE UNIVERSITY
NEW HAVEN, CT 06510

SCIENTIFIC REVIEW OFFICER

VOSVICK, MARK A, PHD
SCIENTIFIC REVIEW OFFICER
CENTER FOR SCIENTIFIC REVIEW
NATIONAL INSTITUTES OF HEALTH
BETHESDA, MD 20892

OBSERVER

RUBERT, MARK P, PHD
SCIENTIFIC REVIEW OFFICER
CENTER FOR SCIENTIFIC REVIEW
NATIONAL INSTITUTES OF HEALTH
BETHESDA, MD 20892

* Temporary Member. For grant applications, temporary members may participate in the entire meeting or may review only selected applications as needed.

Consultants are required to absent themselves from the room during the review of any application if their presence would constitute or appear to constitute a conflict of interest.
